# Upper Extremity Deep Vein Thrombosis Secondary to Gastric Cancer: A Case Report

**DOI:** 10.7759/cureus.106625

**Published:** 2026-04-08

**Authors:** Maimi Takano, Takuya Otsuki, Kosuke Ishizuka, Iori Motohashi, Kenya Ie, Chiaki Okuse

**Affiliations:** 1 Department of General Internal Medicine, St. Marianna University School of Medicine, Kawasaki, JPN; 2 Department of General Internal Medicine, Kawasaki Municipal Tama Hospital, Kawasaki, JPN; 3 Department of General Medicine, Yokohama City University School of Medicine, Yokohama, JPN

**Keywords:** cancer-associated thrombosis, deep vein thrombosis, gastric cancer, trousseau syndrome, upper extremity thrombosis

## Abstract

Upper extremity deep vein thrombosis (UEDVT) is uncommon and is frequently secondary to malignancy, central venous catheterization, or hypercoagulable states. Cancer‑associated thrombosis represents a major cause of morbidity and mortality in patients with malignancy. A 75‑year‑old Japanese woman presented with swelling of the left upper extremity accompanied by epigastric discomfort. Contrast‑enhanced computed tomography revealed thrombosis in the left subclavian vein and multiple metastatic lesions, including hepatic hilar lymph node and spinal metastases. Esophagogastroduodenoscopy demonstrated irregular mucosa with erosions from the gastric angle to the antrum, and pathological examination confirmed poorly to moderately differentiated adenocarcinoma with signet‑ring cell features. The patient was diagnosed with UEDVT secondary to gastric cancer. Anticoagulation therapy with unfractionated heparin improved swelling and pain, and the patient was transitioned to oral apixaban. Chemotherapy was subsequently initiated; however, the patient died nine months after diagnosis due to disease progression. This case highlights the importance of investigating occult malignancy in patients presenting with unexplained venous thromboembolism and initiating prompt anticoagulation therapy.

## Introduction

Deep vein thrombosis (DVT) most commonly affects the lower extremities, whereas upper extremity involvement accounts for only a small proportion of cases [1]. Upper extremity DVT (UEDVT) may be primary or secondary, with secondary causes, including intravascular devices, coagulation abnormalities, and malignancy [1,2]. Cancer-associated VTE is often referred to as Trousseau syndrome and represents a clinically significant complication that worsens prognosis [3]. Malignancy promotes thrombosis through several interacting mechanisms, including tumor cell procoagulant activity, tissue factor expression, inflammatory cytokine release, endothelial injury, and activation of platelets and the coagulation cascade [4]. Although UEDVT is less common than lower extremity DVT, it is clinically important because it may be the first manifestation of an occult malignancy. Its diagnosis may also be challenging because the presentation is less typical than that of lower extremity DVT, and imaging evaluation of central upper extremity veins, particularly the subclavian vein, may be technically limited [5]. Therefore, careful evaluation for occult cancer is essential when unexplained thrombosis is identified. Here, we report a case of gastric cancer initially detected following presentation with UEDVT [3].

## Case presentation

A 75-year-old Japanese woman presented with left upper extremity swelling. She had experienced epigastric discomfort for 1 month, and 10 days before admission, developed swelling and pain in the left side of her neck and shoulder. Her medical history was unremarkable. Physical examination showed a body temperature of 35.9°C, heart rate of 101 beats per minute, blood pressure of 141/75 mmHg, and respiratory rate of 16 breaths per minute. Swelling was observed from the left upper arm to the forearm with jugular venous distention. Laboratory tests showed an elevated D-dimer level of 13.7 μg/mL (Table 1).

**Table 1:** Laboratory findings on admission APTT, activated partial thromboplastin time; CRP, C-reactive protein; HbA1c, hemoglobin A1c; PT-INR, prothrombin time-international normalized ratio.

Variables	On admission	Reference range	Unit
White blood cell count	5.5	3.3-8.6	×10³/μL
Red blood cell count	4.07	4.35-5.55	×10⁶/μL
Hemoglobin	12.5	13.7-16.8	g/dL
Hematocrit	38.0	40-52	%
Platelet count	27.6	15-35	×10⁴/μL
Neutrophils	70.1	40-70	%
Lymphocytes	21.9	20-50	%
Monocytes	6.5	2-8	%
Eosinophils	0.3	0-5	%
D-dimer	13.7	<1.0	μg/mL
PT-INR	0.94	0.85-1.15	
APTT	29.8	25-35	sec
Fibrinogen	540	200-400	mg/dL
Total bilirubin	0.6	0.2-1.2	mg/dL
Aspartate aminotransferase	25	13-30	U/L
Alanine aminotransferase	16	10-42	U/L
Lactate dehydrogenase	253	124-222	U/L
Alkaline phosphatase	351	106-322	U/L
Creatine kinase	209	59-248	U/L
Total protein	6.9	6.6-8.1	g/dL
Albumin	3.9	4.1-5.1	g/dL
Blood urea nitrogen	13.3	8-20	mg/dL
Creatinine	0.67	0.65-1.07	mg/dL
Sodium	141	138-145	mEq/L
Chloride	104	101-108	mEq/L
Potassium	4.1	3.6-4.8	mEq/L
Glucose	116	70-109	mg/dL
HbA1c	6.2	4.6-6.2	%
CRP	0.13	<0.14	mg/dL

**Figure 1:**
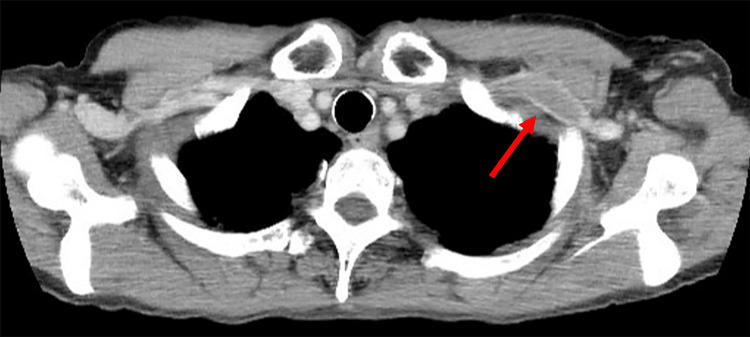
Contrast-enhanced CT. Contrast-enhanced axial CT of the upper chest showing a filling defect in the left subclavian vein (red arrow), consistent with thrombosis.

Contrast-enhanced computed tomography (CT) revealed thrombosis of the left subclavian vein, hepatic hilar lymph node metastasis and spinal metastasis (Figure 1), and peritoneal lymph node swelling.

**Figure 2:**
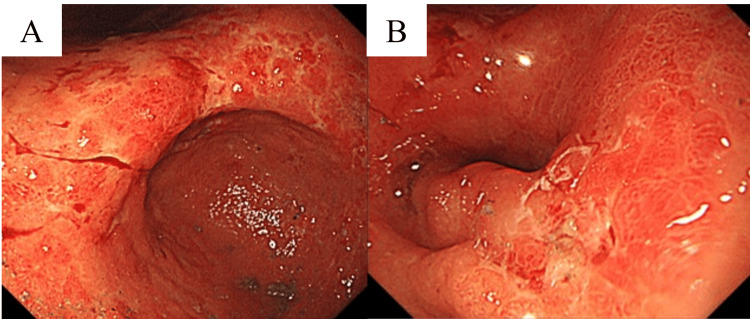
Esophagogastroduodenoscopy demonstrating abnormal mucosa extending from the gastric angle to the antrum. (A) Endoscopic view of the gastric angle showing irregular erythematous mucosa with superficial erosions. (B) Endoscopic view of the antral region showing persistent mucosal irregularity with poor distensibility of the involved segment. These findings were suspicious for gastric malignancy.

Esophagogastroduodenoscopy revealed irregular mucosa with erosions in the region from the gastric angle to the antrum, along with poor distensibility in the same area (Figures 2A, 2B).

Pathological findings confirmed poorly to moderately differentiated adenocarcinoma with signet ring cell features, which led to a diagnosis of UEDVT secondary to gastric cancer. The patient was initiated on anticoagulation therapy with unfractionated heparin, resulting in improvement of swelling and pain in the left upper extremity by day 9. She was transitioned to oral apixaban (20 mg/day) and subsequently discharged. Chemotherapy was initiated based on a diagnosis of gastric cancer. The patient died nine months after the initial diagnosis due to progressive deterioration of her condition.

## Discussion

The diagnostic approach in this case was based on previously described algorithms for suspected UEDVT, which recommend sequential assessment using clinical probability, D-dimer testing, and compression ultrasonography as the initial imaging modality [6,7]. In the present case, the presence of unilateral upper extremity swelling, neck swelling with jugular venous distention, and markedly elevated D-dimer level suggested a moderate to high clinical probability of UEDVT. According to these algorithms, D-dimer is primarily used as a rule-out test in patients with low clinical probability, whereas patients with moderate-to-high suspicion should undergo direct imaging [6,7]. Although compression ultrasonography is generally recommended as the first-line imaging test, its diagnostic accuracy may be limited for evaluating central veins such as the subclavian and brachiocephalic veins, and inconclusive or false-negative results have been reported [8]. Furthermore, acoustic shadowing from the clavicle may hinder adequate visualization and compression of these veins, reducing diagnostic sensitivity [8]. At our institution, scheduling compression ultrasonography performed by experienced vascular technologists may require approximately one week. Considering this potential delay, together with the possibility of false-negative ultrasonography results for central venous thrombosis, contrast-enhanced CT, which was immediately available, was selected to confirm thrombosis and simultaneously evaluate for possible underlying malignancy. In this clinical context, the markedly elevated D-dimer level supported the suspicion of VTE but was not used as a standalone diagnostic test. This is consistent with previous reports indicating that D-dimer has high sensitivity but limited specificity and should be interpreted in combination with clinical probability and imaging studies [6,7].

In this case, anticoagulation was initiated with unfractionated heparin and subsequently transitioned to apixaban. Initial use of unfractionated heparin allowed rapid anticoagulation with dose flexibility during the acute phase. After clinical stabilization, apixaban was selected because direct oral anticoagulants (DOACs) are considered an effective alternative to low-molecular-weight heparin (LMWH) for cancer-associated thrombosis (CAT) in appropriately selected patients [6]. Recent evidence suggests that DOACs and LMWH have comparable efficacy in cancer-associated VTE, and treatment choice should be individualized based on bleeding risk, cancer type, and patient characteristics [6]. In patients with active or metastatic cancer, anticoagulation is generally recommended for at least three to six months and continued while the malignancy remains active [6].

Interventional strategies have also been investigated. Saricaoglu et al. reported that catheter-directed thrombolysis improved thrombus resolution and symptoms compared with anticoagulation alone in selected patients with DVT, although treatment should be individualized according to clinical severity and bleeding risk [9]. However, because our patient had cancer-associated UEDVT without limb-threatening ischemia and showed prompt improvement with anticoagulation, conservative anticoagulant therapy was considered appropriate.

Primary DVT of the upper extremity is rare, while secondary DVT is more common and associated with the placement of intravascular devices, coagulation abnormalities, and malignancy [1,2]. DVT secondary to malignancy, known as Trousseau syndrome, is referred to as cancer-associated VTE [3]. DVT of the upper extremity accounts for 5-10% of all cases of DVT [1]. UEDVT differs from lower extremity DVT in that local mechanical or iatrogenic factors, particularly central venous catheters and pacemaker leads, play a more prominent etiologic role, whereas lower extremity DVT is more often associated with immobilization, surgery, or trauma [6]. Primary UEDVT is rare, with an estimated annual incidence of 1-2 cases per 100,000 population [1]. Most cases of UEDVT are secondary, and the most common causes include central venous catheters (e.g., central venous lines and pacemakers), which account for 7-41% of cases [10]. Non-catheter-related UEDVT is also clinically important because it may reflect underlying hypercoagulable states such as malignancy or thrombophilia [6]. Hypercoagulable states (e.g., thrombophilia and malignancies) are also associated with secondary UEDVT [10]. In this case, VTE was identified in the subclavian vein. Because no intravascular device was present, this case was classified as non-catheter-related secondary UEDVT associated with malignancy. Subclavian vein involvement is clinically important because it represents central venous thrombosis and may cause prominent arm swelling, neck swelling, and venous distention. Furthermore, evaluation of the subclavian vein by ultrasonography may be limited by acoustic shadowing from the clavicle, which can reduce diagnostic sensitivity [8]. UEDVT may also be complicated by pulmonary embolism (PE), although the incidence appears to be lower than in lower extremity DVT; therefore, prompt diagnosis and treatment remain essential [6]. Although data are limited, Mahajan et al. suggest that the site of thrombosis may vary depending on the type of cancer (e.g., breast, prostate, lung, colorectal, non-Hodgkin lymphoma, bladder, uterine, kidney, pancreatic, stomach, ovarian, brain, and myeloma) [11]. The typical patterns observed include PE±DVT in 58% of cases, proximal DVT only in 22%, isolated distal DVT in 11%, and unspecified lower extremity DVT in approximately 8% [11]. The site of thrombosis may differ based on the primary cancer site. For instance, PE±DVT is more common in lung (71%), thoracic (61%), and stomach cancers (60.8%), while proximal DVT is slightly more frequent in bladder cancer (34%) [11]. Proximal DVT, including UEDVT, is relatively common, partly due to the increased use of central venous catheters, which elevate the risk of DVT [12]. VTE is commonly associated with gastric and pancreatic cancers, and the risk of VTE is highest in metastatic disease, followed by locally advanced cancer, and then cancer is confined to the primary site [13]. Patients with cancer are known to have a 12-fold higher risk of VTE compared with patients without cancer [14]. Furthermore, over 60% of patients with CAT die within one year of diagnosis [14], highlighting its significant clinical impact. In this case, the patient had Stage IV gastric cancer and died nine months after diagnosis. In patients with CAT, anticoagulant treatment is critical. The duration of anticoagulant therapy is typically three to six months and should be continued as long as the cancer remains active [15]. Active cancer is defined as metastatic disease or ongoing cancer treatment (chemotherapy and/or radiation therapy). Conversely, cancer in remission for more than six months is considered inactive [15]. Patients with CAT often have advanced-stage cancer and a poor prognosis. When encountering unexplained VTE, it is essential to conduct a thorough search for cancer as a potential underlying cause and initiate anticoagulant therapy concurrently. In a large population-based analysis, patients with gastric cancer who developed VTE had significantly worse survival, with median overall survival of approximately 4 months compared with 10 months in those without VTE [16]. Furthermore, VTE has been reported as an independent prognostic factor in gastric cancer. In multivariate analysis, the presence of VTE was independently associated with increased mortality even after adjustment for disease stage and other clinical variables [17]. Metastatic gastric cancer itself carries a poor prognosis, and thrombotic complications are more common in advanced-stage disease [18]. In this context, the present patient’s survival of nine months after diagnosis is consistent with previously reported outcomes in metastatic gastric cancer complicated by thrombosis and supports the interpretation that VTE reflects advanced disease burden and adverse prognosis.

## Conclusions

We report a case of UEDVT that led to the diagnosis of advanced gastric cancer. Unexplained VTE may represent the initial manifestation of malignancy. Prompt diagnostic evaluation for occult cancer and initiation of anticoagulant therapy are essential for appropriate management and may influence prognosis.
